# Advances in Pathogenesis and Treatment of Skin Cancer

**DOI:** 10.3390/ijms26031255

**Published:** 2025-01-31

**Authors:** Enrica Flori, Giorgia Cardinali, Vittoria Maresca

**Affiliations:** Laboratory of Cutaneous Physiopathology and Integrated Center of Metabolomics Research, San Gallicano Dermatological Institute, IRCCS, 00144 Rome, Italy; enrica.flori@ifo.it (E.F.); vittoria.maresca@ifo.it (V.M.)

This Special Issue is a collection of papers on skin cancers, focusing on their etiopathogenesis and the most innovative and effective therapies. The incidence of melanoma and non-melanoma skin cancers is steadily increasing, representing a significant public health concern. In recent years, significant efforts have been made to develop effective therapeutic options, although many questions remain open. 

Gosman et al. [1] wrote a comprehensive review of the literature on cutaneous melanoma and its management. Intermittent exposure to ultraviolet radiation (UVR) and childhood sunburn are important pathogenetic factors for this neoplasm, especially in subjects with a fair complexion. Pale skin associated with a high number of nevi indicates the presence of a low level of melanin, the main physical and chemical filter against UVR. Hereditary predisposition is another risk factor for melanoma. Several high-penetrance genes such as *CDKN2A, CDK4,* and *BAP1* are the most commonly mutated in hereditary melanoma. Some medium-penetrance genes, such as *MC1R*, which encodes the alpha-melanocyte-stimulating hormone receptor, are also involved in inherited cutaneous melanoma. The synthesis of a good photoprotective pigment, eumelanin, is recognized as a guarantee of photoprotection. Red-haired individuals are often carriers of MC1R polymorphisms, commonly known as loss-of-function variants. The paper by Cerdido et al. [2] belongs to a research group that has consolidated experience in the biology of MC1R and its ligand, αMSH. The authors consider the attribute "loss of function" to be an inappropriate definition. The same research group previously described for the first time the ability of stimulated MC1R to activate mitogenic extracellular signal-regulated kinase 1 and 2 (ERK1/2) signaling [3]. ERK1/2 is the effector of the MAPK pathway, which is primarily involved in determining the aggressiveness of melanoma. Here, the authors present an interesting study using a human melanoma line to create a construct that allows the comparison of the transduction efficacy of two polymorphic variants of MC1R associated with the red hair phenotype. The authors demonstrate minimal residual activity concerning cAMP-related signaling, but highlight effective transduction for the ERK pathway. This result means that the functionality of MC1R in the presence of these polymorphic variants is an aggravating factor in melanoma, increasing its aggressiveness and antioxidant self-defense. Nevertheless, there are cutaneous melanomas, such as acral melanoma, that occur in unexposed areas of the skin. The review by Choi et al. [4] is very comprehensive in this respect. In fact, acral melanoma mainly occurs in dark-skinned individuals and is localized to non-photoexposed areas of the skin (palms and soles). Acral melanoma is a challenge from a biological point of view because it is an extremely aggressive form of melanoma, but above all, it is a social challenge because it occurs in developing countries, on darker skin (where melanoma is generally less common), and prevention education is less discussed and disseminated. It is also clear that staging and therapeutic approaches are more complex for socio-economic reasons.

Melanoma has a good chance of complete resolution if surgically removed in the radial growth phase. However, once the tumor has spread from the epidermal layers into the dermal compartment, it rapidly evolves and becomes very aggressive with a lethal outcome. Two decades ago, the identification of the somatic *BRAF*^v600E^ mutation (present in approximately 50% of diagnosed melanomas) initiated a new era in the treatment of metastatic melanoma with the introduction of molecular targeted inhibitors (BRAF/MEK inhibitor therapy). Over time, these inhibitors have also shown their limitations, with cases of relapse. However, targeted therapy is a milestone and a very powerful tool, and new studies are now proposing the use of these inhibitors in combined therapy protocols [5]. The real turning point in the treatment of metastatic melanoma has come with immunotherapy protocols, which have shown the ability to cure 48% of metastatic melanoma. Immunotherapy is an ingenious approach that exploits the immunogenicity of melanoma and is now the most encouraging prospect, but much work remains to be carried out for melanomas that do not respond to this approach [6]. This is the challenge of the future and the most arduous. However, there is potential in the use of agents used in an adjuvant modality. In this regard, Lengyel et al. [7] presented an updated retrospective analysis of the efficacy of therapeutic protocols for metastatic melanoma. These include chemotherapy, immune checkpoint inhibitors, BRAF/MEK inhibitor therapy, and combinations thereof. The authors compare the efficacy of different approaches based on clinical parameters. They conclude that combination therapies in metastatic melanoma are associated with a high risk of adverse events and that healthcare professionals need to be able to communicate these important aspects to patients in advance. Healthcare providers need to be aware of how serious a particular approach is in terms of distress. The choice is a balance between prolonging disease-free time and maintaining quality of life. The authors emphasize that triple therapy should not be used as first-line treatment for BRAF-mutant melanoma because it is not well tolerated and further studies are needed to identify the potential population that could benefit from this approach. 

With the increasing incidence of malignant melanoma, new prognostic biomarkers have become increasingly important in the clinical decision-making process. A single biomarker is rarely sufficient to stratify melanoma patients into specific risk groups. Rather, a multi-modal approach is required. The ecto-NOX disulfide-thiol exchanger 2 (ENOX2) protein is a member of the NAD(P)H oxidase family and is associated with cell proliferation and induction of epithelial–mesenchymal transition, as well as cell migration and invasiveness [8]. The work by Böcker and co-workers [9] focuses on the role of ENOX2 as a potential prognostic marker and therapeutic target in malignant melanoma. ENOX2 is particularly attractive as a biomarker because it can be detected in readily available samples such as serum and urine. The authors found that high ENOX2 expression in melanoma was associated with significantly reduced overall, disease-specific, and metastasis-free survival, suggesting its suitability as a complementary prognostic biomarker. Furthermore, low levels of tumor-infiltrating lymphocytes (eTILs) were associated with high ENOX2 expression. In addition, inhibition of ENOX2 by the ENOX2 inhibitor PXD offers a promising therapeutic option in combination with targeted therapies by reducing BRAF inhibitor (BRAFi)-induced AKT activation and thus preventing the survival of cell populations that may develop BRAFi resistance. 

The interesting paper by Foda and Neubig [10] deals with the problem of melanoma cases characterized by intrinsic or acquired resistance to immune or targeted monotherapies [11]. The development of preclinical models that deepen the knowledge of the mechanisms underlying resistance to treatments is a real need for the development of innovative and more effective combination therapies. Small GTPases (Rho and Rac) play a key role in the regulation of the cell cytoskeleton and gene transcription. In up to 50% of melanoma cases, drug resistance is due to increased Rho activity. This leads to increased nuclear translocation of its downstream transcriptional coactivators (myocardin-related transcription factors, MRTF-A/B) [12]. The Rho/MRTF-A pathway has been shown to play a key role in drug resistance in human melanoma. It promotes cell proliferation and migration, and thus tumor progression and metastasis. The contribution of the work by Foda and Neubig was the generation of murine melanoma cell lines resistant to the BRAF^v600E^ -selective inhibitor, vemurafenib, which showed enhanced activation of Rho and MRTF. The authors demonstrated that co-treatment of these cells with Vemurafenib and CCG-257081, an inhibitor of Rho/MRTF signaling, not only reduced their viability but also increased their sensitivity to Vemurafenib treatment. In addition, a vemurafenib-resistant mouse melanoma model has been described for the first time in this study. The development of such models is fundamental to understanding the mechanisms that regulate drug resistance and to exploring the efficacy of combined treatments with targeted and immunotherapies. 

There is increasing evidence that the pathogenesis of melanoma is also associated with specific aberrant epigenetic modifications that lead to drug resistance, immune evasion, apoptosis, and increased metastatic potential. Therefore, a promising therapeutic approach appears to be the reversal of an abnormal epigenetic landscape [13]. An important target for epigenetic therapy in melanoma is EZH2, which is involved in disease progression by negatively regulating a number of tumor suppressor genes involved in cell transformation, proliferation, senescence escape, and apoptosis [14]. In this context, Anestopoulus and co-workers [15] aimed to characterize, in an experimental model of malignant melanoma cells, the therapeutic efficacy of the epigenetic drug tazemetostat (TAZ), a potent and selective EZH2 inhibitor. Its use as both a single agent and in combination with several isothiocyanates (ITCs) was examined. ITCs are natural bioactive phytochemicals reported to exert anticancer activity through the induction of cell cycle arrest and apoptosis, as well as the inhibition of angiogenesis and metastasis, by modulating aberrant epigenetic pathways. By combining TAZ with different ITCs, the authors were able to enhance the anti-melanoma activity of TAZ by mediating the apoptotic response, maintaining its specificity and therapeutic efficacy against human malignant melanoma cells while minimizing toxicity to non-tumorigenic neighboring keratinocytes. Furthermore, the authors documented the effect of specific combined experimental protocols in altering the expression of proteins involved in epigenetic pathways, suggesting that the combination of synthetic epigenetic inhibitors with bioactive phytochemicals may represent a promising alternative approach to the management and treatment of melanoma by restoring a normal epigenetic landscape. 

Another important aspect is the integration of studies from veterinary and human oncology. In this context, it has been reported that there is a high degree of similarity between human and canine melanoma in terms of histology, genetic characteristics, and response to therapy [16]. The review by He et al. [17] provided a comprehensive collection of in vitro studies, animal models, and clinical trials of targeted therapies used in canine malignant melanoma. Canine melanoma is characterized by an abnormal proliferation of melanocytes and, when localized in the oral cavity, is particularly aggressive and often metastatic. Canine oral melanoma shares a high degree of homology with human malignant melanoma in terms of aggressiveness, resistance to conventional therapies, and the molecular pathways involved [18]. This review selected 30 studies from 1976 to 2024, grouped according to the different therapeutic approaches (immunotherapies, small molecule signaling inhibitors, indirect kinase inhibitors, microRNA). All the findings on responses to targeted therapies in dogs contribute to veterinary medicine, but can also be very useful to inspire further innovative strategies in the treatment of human cancers.

In addition to malignant melanoma, skin cancer also encompasses the group of non-melanoma skin cancers (NMSCs), which include basal cell carcinoma (BCC) and cutaneous squamous cell carcinoma (CSCC) [19]. The incidence of CSCC is increasing and although the prognosis is often favorable, in 4% of cases the tumor develops metastases to lymph nodes or distant sites. Angiogenesis and lymphangiogenesis are two events critically involved in tumor progression and dissemination and are therefore correlated with poor prognosis and survival [20]. Vascular endothelial growth factor (VEGF) and its receptors (VEGFR2/3) are expressed in the endothelial cells of blood vessels and are mainly involved in cell growth and vascular lymphatic permeability. The aim of the study by Garcia-Perez et al. [21] was to evaluate the gene expression in primary tumors of key biomarkers associated with angiogenesis and lymphangiogenesis that may have prognostic value. The authors determined the gene expression profile of 49 CSCC samples and evaluated the correlation with tumor progression and disease-free survival. The results reported in their study show that a high expression of vascular endothelial growth factor C (VEGFC) at both the gene and protein levels is closely associated with tumor growth and perineural invasion, as well as reduced disease-free survival.

The review by Nicoletti and co-workers [22] highlights the existing research supporting the theory of a possible link between the pathogenesis of basal cell carcinoma (BCC) and a dysembryogenic process, with a particular focus on the Hedgehog signaling pathway, which is crucial for embryonic development. There is a statistically significant correlation between the topographic distribution of BCC and the embryonic fusion lines of the face and neck, for both onset and spread. Several observations suggest a possible reactivation of embryonic program in BCC pathogenesis: BCC arises from basal cells, which possess stem cell-like properties and play a key role in embryonic skin development; some studies have reported the expression of embryonic markers in BCC cells; mutations in genes involved in the Hedgehog signaling pathway are commonly found in BCC; and finally, the microenvironment surrounding BCC resembles that of developing tissues. Elucidating the precise mechanisms underlying the link between BCC and embryonic development could promote advances in the clinical management of this disease, both in preventing it by highlighting certain anatomical sites at risk of developing this neoplasm and in suggesting innovative targeted molecular therapies. 
